# Prompting Paramedics: The Effect of Simulation on Paramedics’ Identification of Learning Objectives

**DOI:** 10.7759/cureus.5362

**Published:** 2019-08-11

**Authors:** Jeremy Hernandez, Eric S Jeong, Teresa M Chan

**Affiliations:** 1 Emergency Medicine, William Osler Health Sciences Centre, Mcmaster University, Brampton, CAN; 2 Emergency Medicine, Schulich School of Medicine and Dentistry, University of Western Ontario, London, CAN; 3 Emergency Medicine, McMaster University, Hamilton, CAN

**Keywords:** paramedicine, paramedic education, learning

## Abstract

Introduction: Simulation has emerged as a useful educational tool for the continued training of paramedics. Practicing paramedics are thought to learn through reflecting on their own actions in practice, and it is hoped that simulation could spur similar reflection, which could then lead to practice change. Despite this, there is limited data on how these practitioners use simulated experiences to set learning objectives. This study aimed to explore how simulation training affects self-identification of learning objectives in emergency medical services (EMS) providers (a.k.a. paramedics).

Methods: Paramedics (primary care and advanced care) participated in a 30-minute simulated learning session. All participants filled out pre-post surveys identifying their own learning objectives immediately before and after the simulation. An inductive qualitative analysis of these responses were conducted by two authors (EJ, TC) using an interpretive description approach, yielding a list of key themes commonly found in the learning objectives. Pre-post learning objectives were individually compared by the level of specificity as determined by the authorship team. Simple descriptive statistics were generated to describe the number of times that the paramedics’ learning objectives became more or less specific, different, or same.

Results: Thirty-five paramedics who completed the simulation and survey were included. Four major themes emerged in the learning objectives: 1) assessment and diagnostic; 2) communication and collaboration; 3) integration of knowledge; and 4) treatment and management. After simulation, the learning objectives became more specific in 6 (17.1%), less specific in 3 (8.6%), different in 22 (62.9%), and remained same in 4 (11.4%).

Conclusion: Simulation training shows promise in refining perceived learning needs. The results from this study offer insight into paramedics’ self-identification of learning objectives and gaps pre-post simulation experiences. Understanding the underlying psychology of paramedics participating in simulation may help educators better understand how to guide reflection and continuous improvement.

## Introduction

Continuing professional development (CPD) is an integral part of lifelong learning that healthcare providers are taught to practice. CPD allows for the enhancement of knowledge and skills, which ultimately improve clinical performance and patient outcomes. Current state of CPD however, is largely characterized by traditional didactic lectures which have been shown to be ineffective in producing changes in practice [1]. Interventions using interaction-based educational methods have shown to be more effective in improving learning than those using didactic methods [2-3]. Other interventions, such as clinical practice feedback, has been met previously with resistance - since feedback about real cases can be perceived as unsafe and possibly threatening [4]. Understanding what continuing medical education (CME) tools are most effective in fostering knowledge retention and application is therefore critical in improving clinical outcomes.

The use of simulation has emerged as a valuable tool in the continued medical training of paramedics and remains a widespread topic of research contributing to CME improvement [5,6-10]. Simulation is often considered a ‘safe space’ in which professionals or trainees can seek to maintain their skills [11-12]. In particular, simulation-based training has been specifically recommended for training in emergency medicine [13] or paramedicine [5]. Simulation-based learning environments provide learners with opportunities to practice their skills and develop clinical confidence in a controlled environment [14]. In simulation scenarios where the cases and goals are known in advance, participants are encouraged to set objectives as a guiding tool to determine if their learning goals have been achieved following the completion of the simulation experience [15]. Simulation, along with the subsequent debriefing and feedback, is rooted in the learning theories of experiential learning and self-reflection. Effective simulation activities are designed from the ground up to promote self reflection. Simulation, therefore, provides a fertile ground for stimulating self-reflection and guided improvement.

Despite the prevalence of simulation in continuing education, there is limited research on how simulated experiences affect the setting of learning objectives and goals. The primary aim of this study was to determine if short simulated experiences could prompt paramedics to modify their self-identified learning objectives. The secondary aim of this study was to identify common themes in the learning objectives set out by paramedics participating in simulated experiences.

## Materials and methods

Participants

Participants of this study consisted of EMS providers undergoing simulation training at a Canadian university as part of the Centre for Paramedic Education (CPER) Annual Practice Review (APR) course. This APR course is an annual event, and all participants had previous experience with simulation either as a paramedic trainee or as part of their ongoing CPD. Participation in the study was voluntary and written informed consent was obtained from all participants. This study was approved by the Hamilton Integrated Research Ethics Board. 

Study design

All participants completed a two-part survey as part of this study. A baseline pre-simulation survey was administered immediately prior to the simulation training, and a post-simulation survey was given immediately following the simulation training. The pre-simulation survey was completed while the participant paramedics were awaiting their annual simulation training event. This pre-simulation survey included questions regarding participant demographics, self-assessment of perceived knowledge/skills, and self-identification of learning objectives pertaining to the targeted simulation training scenario. The post-simulation survey consisted of the same questions as the pre-simulation survey, with additional questions to determine if they felt their own learning objectives were achieved after completing the simulation training.

Data analysis

Survey responses were de-identified then recorded onto a Microsoft Excel spreadsheet. De-identified survey responses were reviewed by the investigatory team members (TC, EJ) to identify themes in the participants’ learning objectives. Based on the theme of each learning objective from pre-simulation to post-simulation, participants were categorized into one of four groups describing the change in specificity of the learning objectives: “more specific”, “less specific”, “same” or “different”. Learning objectives were considered to have become “more specific” if the post-simulation learning objectives themes remained the same as the pre-simulation, but modified with more granular descriptions (e.g., talked about a smaller portion of a broader topic). Learning objectives were considered to have become less specific if the post-simulation learning objectives themes remained the same as the pre-simulation, but modified with more general descriptions or specifications. Learning objectives were considered to be “same” if post-simulation learning objectives remained unchanged from the pre-simulation learning objectives. If the post-simulation learning objectives themes were different than the pre-simulation learning objectives, they were classified under “different”. Simple descriptive statistics were used to describe the participant demographics and the changes in participants' learning objectives from pre-simulation to post-simulation.

## Results

A total of 44 paramedics participated in the simulation training. Nine participants did not complete the survey and were lost to follow-up, resulting in a study sample of 35. The demographics of the study participants are described in Table 1.

**Table 1:** Demographics of the participants SD = Standard deviation

Demographic Descriptor	Value
Mean age (SD)	36.8 (9.1)
Gender (% female)	59 (41%)
Level of training (n)	ACP (10), PCP (34)
Mean number of years of service (SD)	10.7 (7.7)
Mean number of years of simulation training experience (SD)	10.7 (6.7)

There were four major themes that were identified in the learning objectives, which included assessment and diagnostic, communication and collaboration, integration of knowledge, and treatment and management. The frequency of each theme in the pre and post-simulation learning objectives is reported in Table 2.

**Table 2:** Major themes identified in pre and post-simulation learning objectives reported by paramedics

	Overall frequency out of 35 respondents (%)		Example Quotes
Theme	Pre-simulation	Post-simulation	
Assessment and Diagnostic	34.3 (12)	42.9 (15)	“Familiarization with patient presentation, learning algorithms, and drug dosages.” “Doing a thorough assessment of patient medications and how they contribute to illness or injury.”
Communication and Collaboration	8.6 (3)	8.6 (3)	“[Participate in] interactive group learning review.” “Better teamwork.”
Integration of Knowledge	42.9 (15)	28.6 (10)	“Practice in a controlled environment of information reviewed today.” “To practice skills and [improve] comfort level.”
Treatment and Management	14.3 (5)	20 (7)	“Recognition of med overdose, hyperkalemia treatment, and management of the altered patient.” “Management of trauma in the elderly and management of unstable patients.”

**Figure 1:**
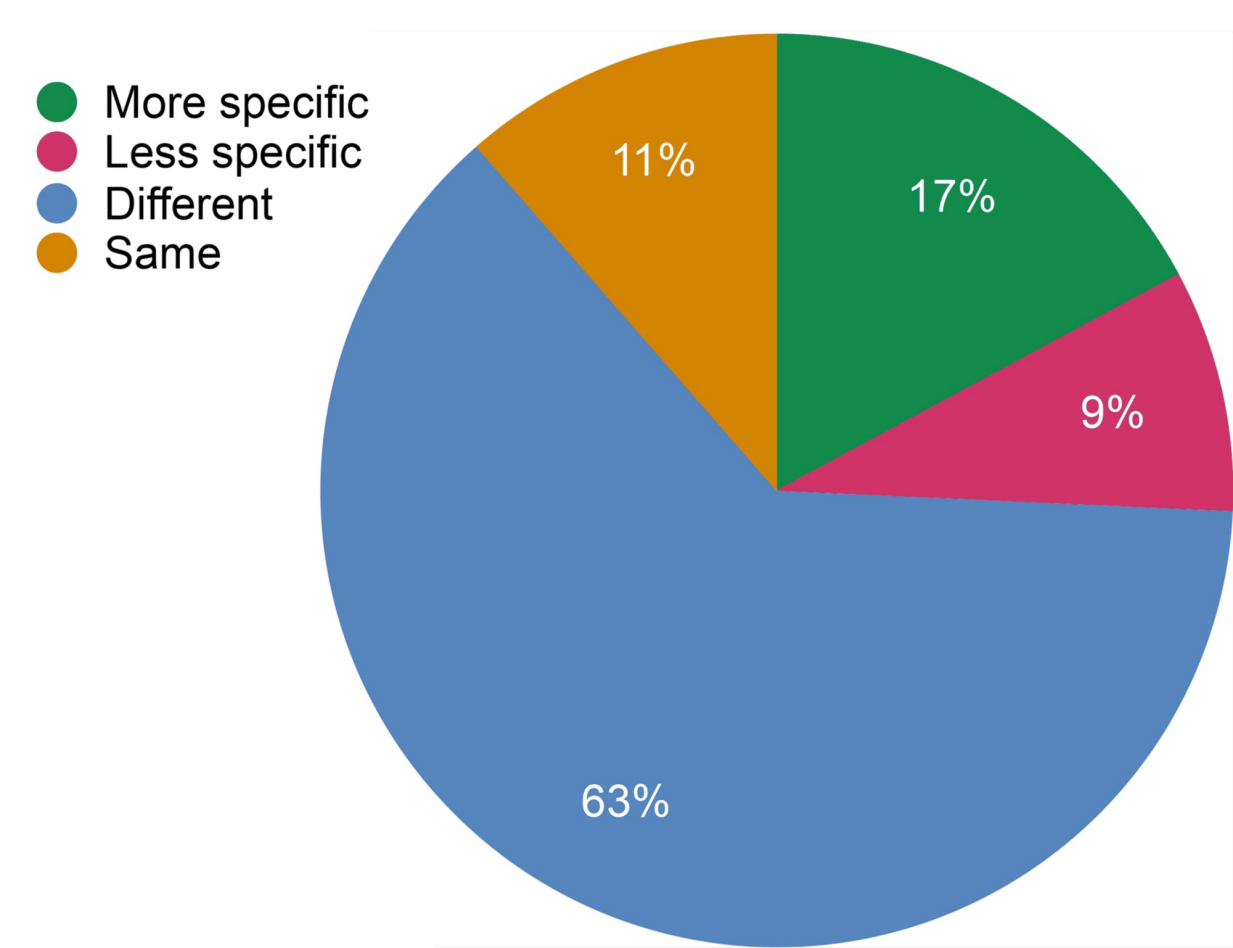
Changes in learning objectives from pre-simulation to post-simulation

After the completion of the simulated training, 63% of the learning objectives became “different”, 17% became “more specific”, 11% became “less specific”, and 9% remained the same. The changes in the specificity of learning objectives from pre to post-simulation is shown in Figure 1.

## Discussion

Simulation is becoming an increasingly common tool for paramedics and emergency medicine trainees to maintain and enhance their skills. Simulation acts to bridge the gap between knowledge acquisition and clinical practice, and actively engages the learner in cognitive, behavioural, and emotional dimensions.

In this study, we investigated how simulation training affects self-identification of learning objectives in EMS providers. Results demonstrated that 89% of participants refined or changed their learning objectives after a simulated learning experience, as indicated by learning objectives which became more specific, less specific, or different. This suggests that simulation training was effective in generating self-reflection. Interestingly, a change in the specificity of learning objective from pre-simulation to post-simulation was observed in 63% of the participants. A possible explanation for this finding is that many participants were unable to set relevant or realistic learning objectives prior to the simulation training, resulting in a change in the learning objective theme. Overall, this suggests that not only does simulation training effectively prompt learners to engage in self-reflection, but also provide insight into the poor baseline ability of professionals to self-assess accurately, as demonstrated in the literature [16].

The secondary aim of this study was to identify major themes in the learning objectives set out by the paramedics before and after the simulated scenario. Participants of simulation training are encouraged to set and define relevant learning objectives that thematically complement the competencies that they desire to improve prior to participating in the simulated scenario. Throughout simulation, participants self-generate feedback which allow them to refine their previous learning objectives so that they can become more relevant for future learning experiences [15]. In this current study, the major themes identified in the learning objectives were: 1) assessment and diagnostic; 2) communication and collaboration; 3) integration of knowledge; and 4) treatment and management.

Prior to the simulation training, integration of knowledge was the most commonly identified competency theme comprising of 42.9% of total participants, followed by assessment and diagnostic (34.3%). After the simulation training, the most common learning objective theme was assessment and diagnostic (42.9%) followed by integration of knowledge (28.6%). This may suggest that a substantial number of participants initially hoped to use the simulation training experience primarily as a means to integrate and apply their knowledge, rather than to acquire knowledge on assessment, diagnosis, and management of the patient. However, during the simulation, it may have been that the experience revealed new knowledge gaps that prompted them to reflect and refine their learning objectives after the simulation.

Findings from this study show promise that simulation training can provide paramedics with improved guidance for self-assessment as well as transfer of learned skills to practice. Learners should be aware that learning objectives identified before simulation training may change post-simulation. Studies show that simulation participants who were better at assessing their own learning were able to retain more information post-simulation and ultimately better equipped to transfer what they learned into another representation [17-18]. Participants should ensure that they use simulation to identify new gaps in their knowledge and understanding.

For educators facilitating simulation training sessions, they should encourage participants to actively reflect by setting learning goals both before and after simulation experiences. Educators may also consider facilitating debriefing sessions to help guide goal setting. Doing so would encourage commitment to change in learners, as suggested in prior studies in the CPD and CME literature [17-18]. It may also be beneficial for simulation curriculum designers to consider how they might cycle back to these post-simulation objectives. Effective curricular integration via targeted spaced repetition [19] may allow simulation educators to harness the true power of simulation for mastery learning [20]. Educators could use the refined, post-simulation learning objectives to redefine the next simulation objective with that same participant. This type of educational practice might help with converting one-off simulations into a form of continuously, personal quality improvement.

## Conclusions

This study suggests that simulation can be a valuable tool for facilitating objective-setting for practicing paramedics, especially when incorporated into a continuing professional development opportunity. Our results show promise that goal setting and identification after simulation can facilitate objectives setting for future practice change. Educators should consider facilitating debriefing sessions, as well as prompting learners to actively set learning goals to encourage commitment to change.
